# Spatial compartmentation and food web stability

**DOI:** 10.1038/s41598-018-34716-w

**Published:** 2018-11-02

**Authors:** Akihiko Mougi

**Affiliations:** Department of Biological Science, Faculty of Life and Environmental Science, Shimane University, 1060 Nishikawatsu-cho, Matsue, 690-8504 Japan

## Abstract

An important goal in ecology has been to reveal what enables diverse species to be maintained in natural ecosystems. A particular interaction network structure, compartments, divided subsystems with minimal linkage to other subsystems, has been emphasized as a key stabilizer of community dynamics. This concept inherently includes spatiality because communities are physically separated. Nevertheless, few theoretical studies have explicitly focused on such spatial compartmentation. Here using a meta-community model of a food web, I show that compartments have less effect on community stability than previously thought. Instead, less compartmentation of a food web can greatly increase stability, particularly when subsystems are moderately coupled by species migration. Furthermore, compartmentation has a strong destabilization effect in larger systems. The results of the present study suggest that spatial limitation of species interactions rather than of community interactions plays a key role in ecosystem maintenance.

## Introduction

According to May, complex ecological communities should not persist because of their inherent instability^1^. However, this theory is clearly lacking to some extent due to the persistence of complex real ecosystems comprised of diverse species and their interactions. Earlier studies have attempted to explain this with non-random interaction network structures, which are not assumed in May’s theory^2–10^. One network structure key for stability is compartments, in which a network is divided into subsystems with minimal linkages to other subsystems^1^. Such compartmentation may create a buffer against ecological perturbations to the whole system because of loose couplings between subsystems^1,11–14^. However, several theoretical studies have shown that strongly compartmented food webs are unstable^15,16^ or show moderate stability only under limited conditions^17^. Importantly, the definition of stability and/or interaction strength can influence predicted status^8,14^. Also, there is little information available regarding compartmentation within food web data, except between major spatially distinct habitats such as pelagic and benthic, shore and offshore, and land and marine^18–22^ (but see also)^23–25^.

It is undeniable that major spatial distinctions between habitats separate an ecological community, creating compartments “among spatially distinct subcommunities^18,20,26–28^”. Despite this, very few studies have examined the role of such major spatial compartmentation in community maintenance. Previous studies focusing on the role of compartments have not explicitly considered space^8,11–17,29^, meaning that some species linking subcommunities are considered to be always globally interacting. In real systems, however, because of spatial distinction, subcommunities would be locally connected by interactions between local populations of some species. Also, such local interactions occurring across a boundary between subcommunities could propagate to each community through migration. This meta-community view^30–34^ provides a different perspective on compartmentation: subcommunities coupled by not only species interactions but also spatial interaction through migration, yet it remains unclear how spatial compartments among spatially distinct subcommunities may affect the maintenance of the whole community.

**Figure 1:**
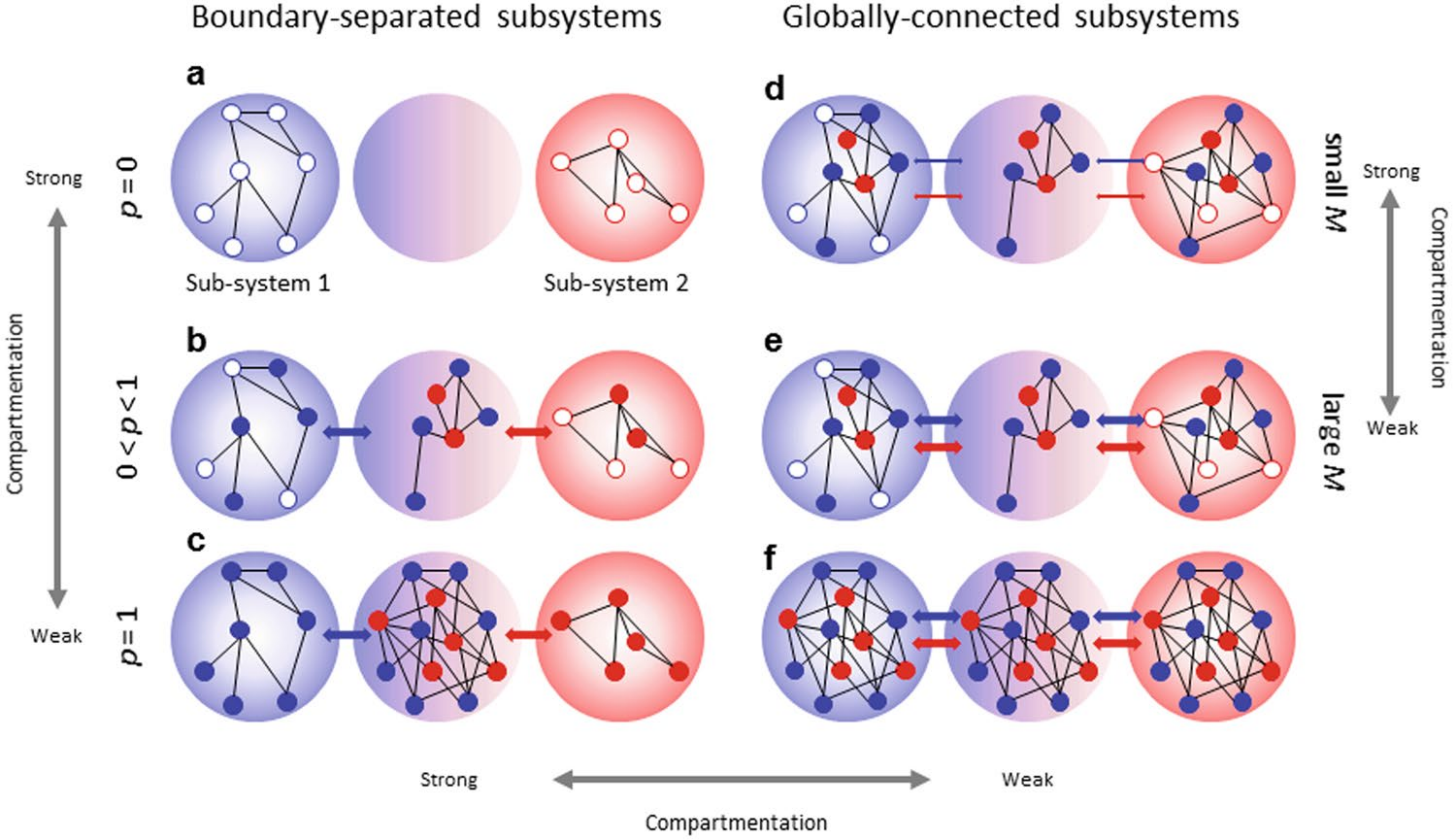
Schematic representation of a spatially compartmented food web model. (**a**–**c**) Boundary-separated subsystems. (**a**,**d**–**f**) Globally-connected subsystems. As *p* decreases, the degree of compartmentation increases. When *p* = 0, subsystems are completely isolated, whereas when *p* = 1, subsystems are completely merged. Each large circle indicates a patch. Large blue and red circles on the left and right, respectively, are the main habitats of species represented by small blue and red circles. The medium circle is a boundary habitat where two subsystems are locally and partially merged by interactions between species migrating from each main habitat. Small blue and red filled circles in each patch indicate local populations of migratory species in different main habitats, whereas open circles of different colors indicate those of non-migratory species. Arrows between patches indicate the degree of species migration. For example, spatial coupling strength in (**d**) is smaller than that in (**e**).

Here, I extend an existing meta-food web model^34^ to examine the effects of spatial compartments on community stability (Methods). In natural communities, species interactions are undoubtedly spatially limited, and local populations of each species interact and move among habitats (Fig. 1f). This meta-community view should also be true on a larger spatial scale. Consider two major spatially distinct habitats and a boundary habitat between them. Each organism can randomly move between these habitats. The proportion of migratory species is controlled by a parameter *p*. When *p* is small, sub-food webs are linked by few interactions between local populations within each sub-food web. At the extreme, sub-food webs are completely divided (*p = *0) (Fig. 1a). By contrast, when *p* is large, sub-food webs are linked by many interactions between local populations within each sub-food web (Fig. 1c). The rate of species migration between local patches is controlled by a scaling parameter that controls spatial coupling strength *M* (Methods). When *M* = 0, sub-food webs are isolated, whereas when *M* is extremely large, sub-food webs are strongly coupled, behaving as a single food web.

In nature, distinct sub-food webs are linked by species interactions between local populations within a local habitat. This linkage might occur in two ways, depending on the degree of penetration of migratory species into external subsystems (penetration degree). First, it may be limited to a local (boundary) habitat (boundary-separated subsystems). In this case, sub-food webs of the main habitats are locally and partially merged by interactions between species migrating between each sub-food web (Fig. 1b,c). Second, the sub-food webs may be linked across whole habitats (globally-connected subsystems). In this case, some species in a sub-food web may pass the boundary habitat and appear in another sub-food web (Fig. 1e,f).

Here, I define “compartmentation” as the degree of spatial segregation between sub-food webs. More specifically, the degree of compartmentation is controlled by three elements: the proportion of migratory species (*p*), spatial coupling strength (*M*), and penetration degree (boundary-separated or globally-connected subsystem). When *p*, *M*, and/or penetration degree are low, compartmentation is expected to be strong (Fig. 1). In the present study, *p*, *M*, and penetration degree are controlled to examine the effects of spatial compartmentation on stability. An index of stability “community stability,” which is estimated based on local stability (the tendency for community composition to return to its original equilibrium after a small perturbation), was used.

I show that, in the sense of spatial compartmentation, compartmentation has less effect on community stability. On the contrary, less compartmentation has more stabilizing effect particularly in smaller systems, although a moderate level of spatial coupling strength is required in larger systems. This supports little evidences of compartmented subcommunities within small systems^18^. Furthermore, large systems with major habitat divisions may be maintained not by limited species interactions between subcommunities, but by a moderate species migration. The present study suggests that spatial limitation of species interactions rather than of community interactions plays a key role in community maintenance.

## Results

**Figure 2:**
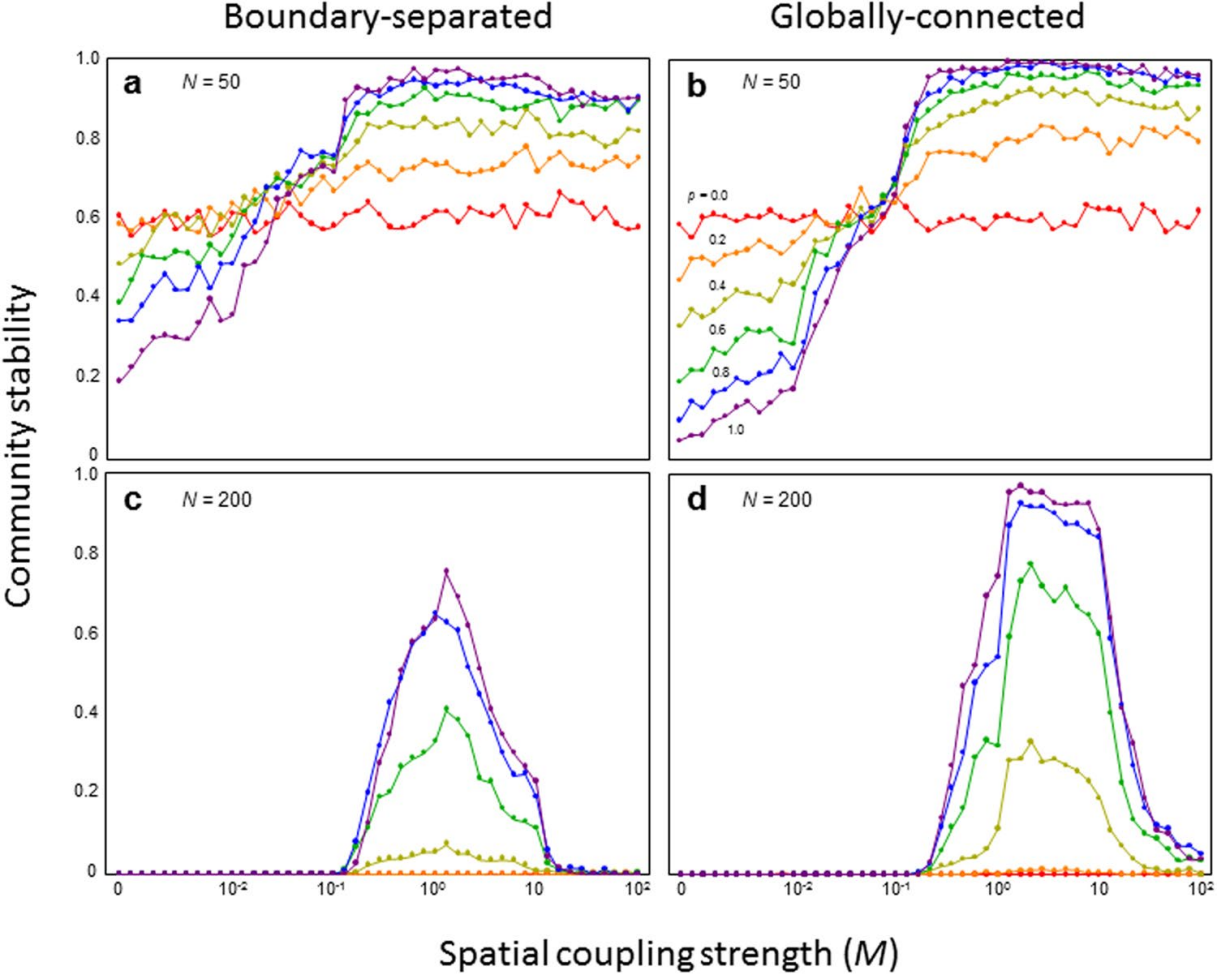
Relationships between spatial coupling strength and stability with varying proportions of migratory species. (**a**,**b**) I assumed *N* = 50. (**c**,**d**) I assumed *N* = 200. (**a**,**c**) Boundary-separated subsystems. (**b**,**d**) Globally-connected subsystems. Colors indicate different levels of *p*. *N* = 50 and *C* = 0.5.

Consider a perfect compartmented food web comprised of *N* species (distinct sub-food webs are comprised of *N*/2 species), any pair of which are connected to each other with probability *C* (connectance), defined as the proportion of realized interaction links of the possible maximum interaction links of a given network model, in which no species move between habitats (*p* = 0; Fig. 1a). In this extreme case, it is trivial that the strength of spatial coupling *M* does not affect community stability (Fig. 2a,b). However, if sub-food webs are coupled by migratory species, the spatial coupling strength *M* dramatically alters the effects of *p* on community stability. When habitats are weakly coupled (smaller *M*), less connected food webs (smaller *p*) have higher stability. In contrast, when habitats are tightly coupled (larger *M*), more connected food webs have higher stability (Fig. 2a,b). Further, more spatially compartmented food webs (smaller *p* and *M*) have lower stability than less spatially compartmented food webs (larger *p* and *M*), regardless of penetration degree. These results were qualitatively unchanged by varying the stability index (Fig. S1), type of network (Fig. S2), or connectance (Fig. S3).

However, system size or species richness can greatly affect the results in four ways. First, if the system becomes extremely large and *M* is large or small, stability can be extremely low or zero regardless of the degree of *p* (Fig. 2c,d). Such a system can, however, be stable within a moderate range of *M*. Note that destabilization is not likely to occur when *M* is large in smaller systems (Figs 2a,b, and S4). Second, when habitats are moderately coupled, the degree of compartmentation can dramatically affect stability. Less compartmented food webs tend to show high stability (Fig. 2c,d). Third, both more (smaller *p* and *M*) and less (larger *p* and *M*) spatially compartmented food webs have extremely low stability. Finally, the difference in stability between boundary-separated and globally-connected subsystems becomes greater the larger the system. Globally-connected subsystems tend to be more stable than boundary-separated ones (Fig. 2c,d).

## Discussion

The results of the present study suggest that in smaller systems, less spatially compartmented food webs in terms of all elements of compartmentation are likely to be stable compared with more compartmented ones. By contrast, in larger systems, the degree of compartmentation becomes more central to stability. Stabilization arises only under a moderate coupling strength; more spatially compartmented food webs will not stabilize, and stability tends to be higher in less compartmented food webs. Taken together, the results indicate that spatial compartmentation has a lesser and more negative effect on community stability in larger systems than in smaller ones.

Less spatially compartmented food webs are more likely to be stable, suggesting that compartments play a less significant role in maintaining food webs than previously thought^15,17^. This is supported by three results: First, in more complex food webs with higher species richness, as in natural ecosystems, compartmentation is less stabilizing because of strong inherent instability. Second, globally-connected sub-food webs can be more stable than boundary-separated ones. Third, sub-food webs weakly coupled by migration are highly unstable. These results suggest that less stable, more compartmented food webs are maintained only by a few highly mobile species, partially supporting the prediction of a spatially implicit food web model that a mobile higher order organism can stabilize food web dynamics when embedded in a variable and expansive spatial structure^29^. An alternative hypothesis is that, in real compartmented food webs, more species may couple sub-compartmented systems through migration than previously recognized.

The role of compartmentation depends on spatial scale. More specifically, compartmentation will become less crucial for ecosystem maintenance at a larger spatial scale. In compartmented food webs on a huge spatial scale (including ocean and continent systems), such large compartments may not work as a stabilizer, whereas the spatial limitation of species interactions within each subsystem (ocean or continent system) may be a key stabilizer for such a large compartmented system. In other words, large compartmented systems are formed by mere coupling of more stable subsystems by some species. This argument may justify research that independently studies each mechanism by which large spatially distinct sub-ecosystems are maintained.

The locality of species interactions and/or weak compartmentation might play a significant role in maintaining food webs. In some systems, many species can move between habitats, building species-rich communities locally (Fig. 1c,e). In contrast, if only a small proportion of species can move, some local communities are species-poor (Fig. 1b,d). May^1^ speculated that inherently stable, local, species-poor systems contribute to the stability of the whole system. However, the present study reveals a completely different result: Species-rich local communities contribute to the stability of the whole community. The least stable communities with the greatest species diversity are most affected by immigration^31,34^. This suggests that the effects of stabilizing self-regulation through migration^34^ outweigh the stabilization effects of species-poor systems.

In conclusion, the present results suggest that in larger systems, spatial compartmentation may not provide stabilization; instead, the spatial limitation of species interactions plays a significant role in stabilizing systems. Although the question of whether this theory is robust to various other stability indices remains open, the present study provides a new perspective on compartments in ecological networks.

## Methods

Consider a meta-food web model (Fig. 1). This model assumes a random (or cascade in Fig. S2) food web in which each pair of species, *i* and *j* (*i*, *j* = 1, …, *N*), are connected by a trophic interaction with probability *C* (connectance), which is defined as the proportion of realized interaction links *L* of the possible maximum interaction links *L*_max_ of a given network model (*L* = *CL*_max_). The maximum link number, *L*_max_, is *N*(*N *− 1)/2. The spatial food web model is defined by the following ordinary differential equation^34,35^:

1 $$\frac{d{X}_{il}}{dt}={X}_{il}({r}_{il}-{s}_{il}{X}_{il}+\sum _{j=1}^{N}{a}_{ijl}{X}_{jl})-M(\sum _{k=1(l\ne k)}^{{H}_{N}}{m}_{ilk}{X}_{il}-\sum _{k=1(k\ne l)}^{{H}_{N}}{m}_{ikl}{X}_{ik}),$$

where *X*_*il*_ (*l* = 1 … *H*_*N*_) (where *H*_*N*_ is the number of patches) is the abundance of species *i* in habitat *l*, *r*_*il*_ is the intrinsic rate of change of species *i* in habitat *l*, *s*_*il*_ is the density-dependent self-regulation of species *i* in habitat *l*, and *a*_*ijl*_ is the interaction coefficient between species *i* and species *j* in habitat *l*. Interaction coefficients are defined as *a*_*ijl*_ = *e*_*ijl*_*α*_*ijl*_ and *a*_*jil*_ = –*α*_*ijl*_, where *α*_*ijl*_ is the consumption rate and *e*_*ijl*_ (<1) is the conversion efficiency. In the present analysis, it was assumed that habitats are heterogeneous and there are no within-species parameter correlations among them. The migration rate is the product of a scaling parameter, *M* (spatial coupling strength), and the species-habitat-specific migration rate, *m*_*ilk*_, where *k* = 1…*H*_*N*_ but *k* $\ne$ *l*. For simplicity, it was assumed that *m*_*ilk*_ = *m*_*ikl*_. Equilibrium species abundance *X*_il_^*^ and parameters *s*_*il*_, *e*_*ijl*_, *α*_*ijl*_, and *m*_*ilk*_ were randomly chosen from a uniform distribution, U[0, 1]. *r*_*il*_ was calculated such that *dX*_*il*_/*dt* = 0 for all *i* and *l*^34,36^.

I assumed a three-patch model (*H*_*N*_ = 3) in which patches 1 and 3 are the original habitats in different sub-food webs and their randomly selected local populations can interact within patch 2 between them. This is the simplest model to examine the effect of spatial compartmentation on community stability. The proportion of migratory species within each community is defined as *p*_*i*_ (*i* = 1 or 3). In the main text, I assumed that *p*_1_ = *p*_3_ = *p* (see Fig. S5 for an example in which this assumption is relaxed). In boundary-separated subsystems (Fig. 1b,c), migration can occur between the original habitat (patch 1 or 3) and boundary habitat (patch 2). In globally-connected subsystems (Fig. 1e,f), migration can occur across all habitats. Given total species number *N*, community sizes in each original habitat are controlled by the proportion of species in sub-food web 1, *q*_1_ (i.e., *q*_3_ = 1 – *q*_1_). Then, *N*_1_ = *Nq*_1_ (*N*_3_ = *Nq*_3_), where *N*_*i*_ (*i* = 1 or 3) is original species number within each original habitat. In the main text, I assumed that *q*_1_ = *q*_3_ = *q* = 0.5 (see Fig. S6 for an example in which this assumption is relaxed). Individual species were randomly assigned to each subsystem and migratory species were randomly chosen in each simulation.

Following earlier studies, I calculated the stability of the systems using a standard local stability analysis based on a Jacobian community matrix^34,36^. Then, I evaluated the community stability, or the probability of local equilibrium stability, which is estimated as the frequency of locally stable systems across 1000 sample communities. Local stability is calculated based on the eigenvalues of the Jacobian matrix. If the real part of the dominant eigenvalue is negative, the system is locally stable; otherwise, it is unstable. If *p* = 0, stability is calculated based on a system with two patches (because the middle patch is empty). I also used another stability index, resilience (engineering resilience), or the rate of recovery of the original equilibrium after a small perturbation, which is determined using the mean magnitude of the real part of the dominant eigenvalue of J across 1000 samples of locally stable communities (Fig. S1).

## Electronic supplementary material

Supplemental figures

## References

[1] May RM (1972). Will a large complex system be stable?. Nature.

[2] Moore JC, Hunt HW (1988). Resource compartmentation and the stability of real ecosystems. Nature.

[3] Pimm SL, Lawton JH, Cohen JE (1991). Food web patterns and their consequences. Nature.

[4] McCann KS (2000). The diversity-stability debate. Nature.

[5] Williams RJ, Martinez ND (2000). Simple rules yield complex food webs. Nature.

[6] Neutel A-M, Heesterbeek JAP, de Ruiter PC (2002). Stability in real food webs: weak links in long loops. Science.

[7] Brose U, Williams RJ, Martinez ND (2006). Allometric scaling enhances stability in complex food webs. Ecol Lett.

[8] Stouffer DB, Bascompte J (2011). Compartmentalization increases food-web persistence. Proc Natl Acad Sci USA.

[9] Allesina S, Tang S (2012). Stability criteria for complex ecosystems. Nature.

[10] Mougi A, Kondoh M (2012). Diversity of interaction types and ecological community stability. Science.

[11] Gardner MR, Ashby WR (1970). Connectance of large dynamic (cybernetic) systems: critical values of stability. Nature.

[12] McNaughton SJ (1978). Stability and diversity of ecological communities. Nature.

[13] Roberts A, Tregonning K (1980). The robustness of natural systems. Nature.

[14] Teng J, McCann KS (2004). Dynamics of compartmented and reticulate food webs in relation to energetic flows. Am Nat.

[15] Pimm SL (1979). The structure of food webs. Theor Popul Biol.

[16] McCann, K. S. *Food Webs*. (Princeton University Press: Princeton, s Jersey, USA: 2011).

[17] Grilli J, Rogers T, Allesina S (2016). Modularity and stability in ecological communities. Nat Commun.

[18] Pimm SL, Lawton JH (1980). Are food webs divided into compartments?. J Anim Ecol.

[19] Winemiller KO (1990). Spatial and temporal variation in tropical fish trophic networks. Ecol Monogr.

[20] Polis GA, Strong DR (1996). Food web complexity and community dynamics. Am Nat.

[21] Krause AE, Frank KA, Mason DM, Ulanowicz RE, Taylor WW (2003). Compartments revealed in food-web structure. Nature.

[22] Melián CJ, Bascompte J (2002). Complex networks: two ways to be robust?. Ecol Lett.

[23] Yodzis P (1982). The compartmentation of real and assembled ecosystems. Am Nat.

[24] Rezende EL, Albert EM, Fortuna MA, Bascomte J (2009). Compartments in a marine food web associated with phylogeny, body mass, and habitat structure. Ecol Lett.

[25] Guimerà R, Stouffer DB, Sales-Pardo M, Leicht EA, Newman ME (2010). Origin of compartmentalization in food webs. Ecology.

[26] Polis, G. A. & Winemiller, K. O. *Food webs: integration of patterns and dynamics*. (Chapman & Hall, New York: 1996).

[27] Huxel GR, McCann K (1998). Food web stability: the influence of trophic flows across habitats. Am Nat.

[28] Post DM, Conners ME, Goldberg DS (2000). Prey preference by a top predator and the stability of linked food chains. Ecology.

[29] McCann KS, Rasmussen JB, Umbanhowar J (2005). The dynamics of spatially coupled food webs. Ecol Lett.

[30] Pillai P, Loreau M, Gonzalez A (2010). A patch-dynamic framework for food web metacommunities. Theor Ecol.

[31] Gravel D, Canard E, Guichard F, Mouquet N (2011). Persistence increases with diversity and connectance in trophic metacommunities. PLoS ONE.

[32] Pillai P, Gonzalez A, Loreau M (2011). Metacommunity theory explains the emergence of food web complexity. Proc Natl Acad Sci.

[33] Plitzko SJ, Drossel B (2015). The effect of dispersal between patches on the stability of large trophic food webs. Theor Ecol.

[34] Mougi A, Kondoh M (2016). Food-web complexity, meta-community complexity and community stability. Sci Rep.

[35] Mougi A (2016). Spatial complexity enhances predictability in food webs. Sci Rep.

[36] Chen X, Cohen JE (2001). Transient dynamics and food-web complexity in the Lotka-Volterra cascade model. Proc R Soc B.

