# Supplementary material for: Spatial compartmentation and food web stability
Source: Sci Rep. 2018 Nov 2;8:16237. doi: 10.1038/s41598-018-34716-w (PMC6215003; doi:10.1038/s41598-018-34716-w)

**Supplementary Information**

# Spatial compartmentation and food web stability

**A. Mougi**

## Supplementary Figures

To examine the robustness of the results in Fig. 2a and 2b, I used different stability index, network topology, connectance, or species richness. First, the stability is evaluated by resilience (engineering resilience), namely, the rate of recovery to original equilibrium after a small perturbation, which is calculated by the mean magnitude of the real part of the dominant eigenvalue of J across 1000 samples of locally stable communities (Fig. S1). Second, cascade model is used as other network type (Fig. S2). In the cascade model, for each pair of species, $i, j = 1,\ldots, n$ with $i < j$, species $i$ never consumes species $j$, whereas species $j$ may consume species $i$. Third, different values of $C$ (Fig. S3) and $N$ (Fig. S4) are considered. Even if I change these factors, we can see that the results in Fig. 2a, b are qualitatively unchanged. However, in larger $N$, larger $M$ tends to decrease the stability than in smaller $N$.

In addition to these changes, I relax strong assumptions of symmetry of parameters. First is the proportions of migratory species in sub-food webs ($p_1 = p_3 = p$ in the main text). As shown in Fig. S5, even if the change of $p_1$ ($p_3$ is fixed) almost do not affect the result. Second is the community sizes of each original habitat, which are controlled by the proportion of species in sub-food web 1, $q_1$ ($q_3 = 1 – q_1$). In the main text, $q_1 = q_3 = q$. As shown in Fig. S6, even if the change of $q_1$ ($q_3$ is fixed) almost do not affect the result.

**Fig. S1**. Relationships between spatial coupling strength and resilience with varying proportions of migratory species. (a) Boundary-separated subsystems. (b) Globally-connected subsystems. Other information is the same as in Fig. 2a, b.

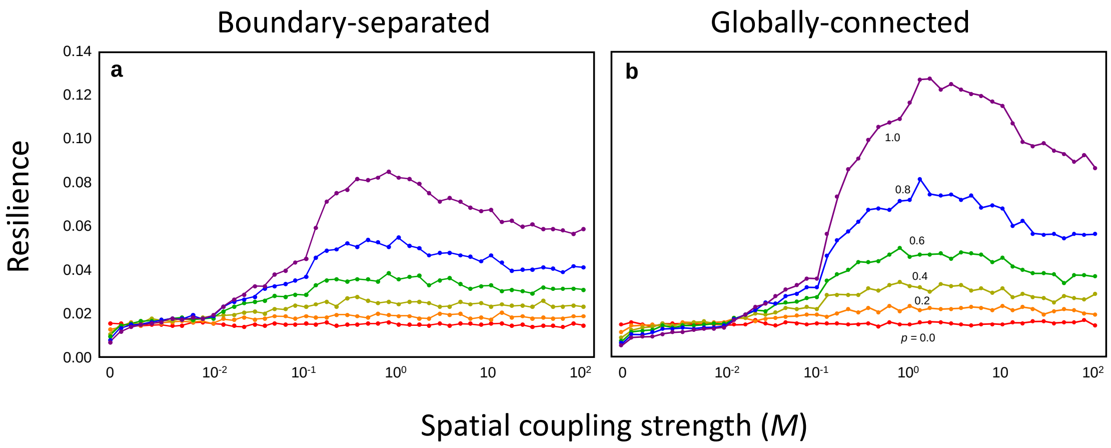

**Fig. S2**. Relationships between spatial coupling strength and stability with varying proportions of migratory species in a cascade model. (a) Boundary-separated subsystems. (b) Globally-connected subsystems. Other information is the same as in Fig. 2a, b.

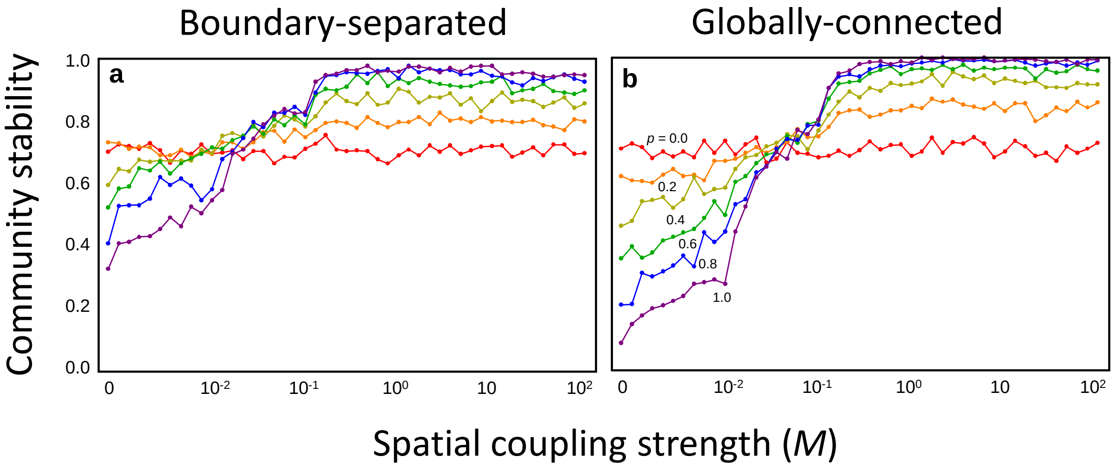

**Fig. S3**. Relationships between spatial coupling strength and stability with varying proportions of migratory species. (a, b) Boundary-separated subsystems. (c, d) Globally-connected subsystems. In (a) and (c), $C$ = 0.75. In (b) and (d), $C$ = 0.25. Other information is the same as in Fig. 2a, b.

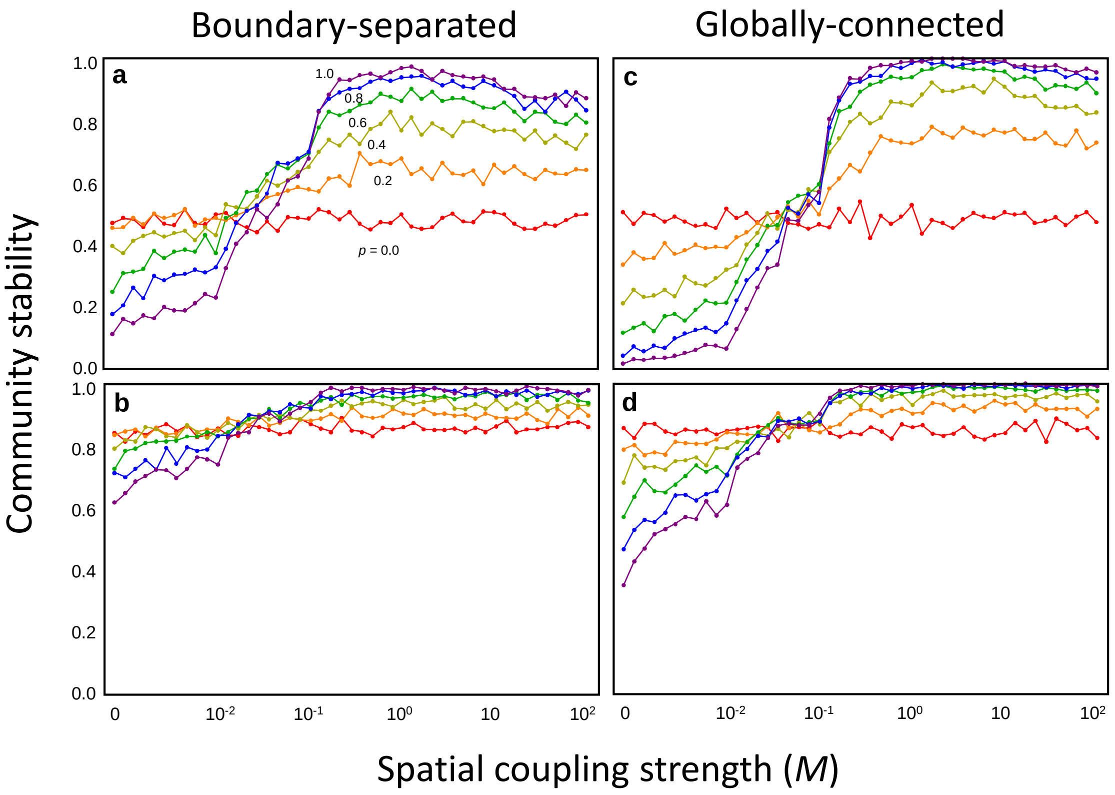

**Fig. S4**. Relationships between spatial coupling strength and stability with varying proportions of migratory species. (a, b) Boundary-separated subsystems. (c, d) Globally-connected subsystems. In (a) and (c), $N$ = 100. In (b) and (d), $N$ = 20. Other information is the same as in Fig. 2a, b. Note that the stability almost does not change even in $M = 10^{10}$.

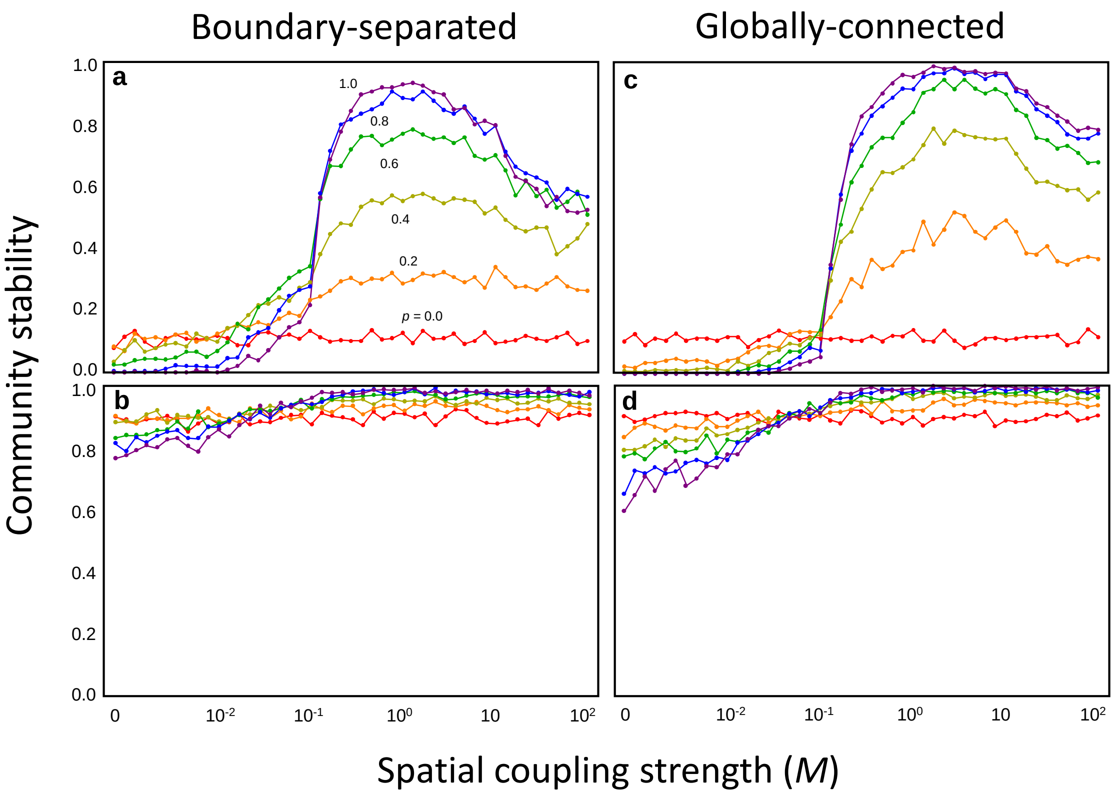

**Fig. S5**. Relationships between spatial coupling strength and stability with varying proportions of migratory species in a patch 1 ($p_1$). (a) Boundary-separated subsystems. (b) Globally-connected subsystems. $p_2$ = 0.5. Other information is the same as in Fig. 2a, b.

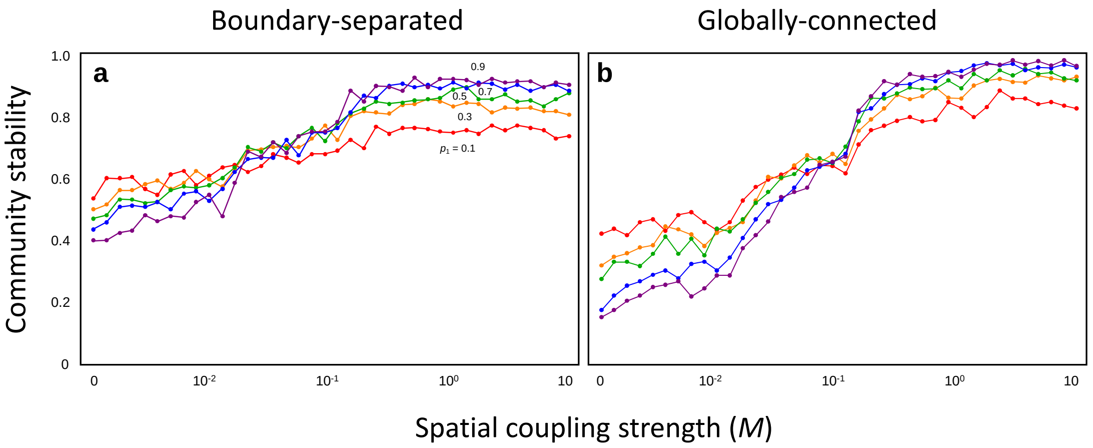

**Fig. S6**. Relationships between spatial coupling strength and stability with varying $q_1$. (a) Boundary-separated subsystems. (b) Globally-connected subsystems. Other information is the same as in Fig. 2a, b.

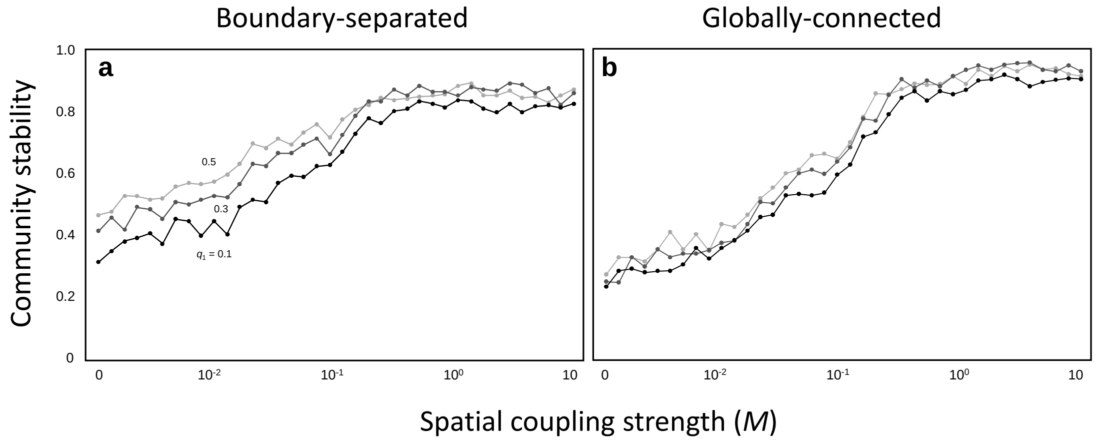

Supplement: Supplementary file 1 — Supplemental figures [file 41598_2018_34716_MOESM1_ESM.pdf]
